# Acute Boric Acid Poisoning With Chronic Renal Failure Successfully Treated With Continuous Hemofiltration

**DOI:** 10.7759/cureus.103795

**Published:** 2026-02-17

**Authors:** Hitoshi Nakajima, Takushi Santanda, Takahiro Shoji, Kazuhiko Sekine

**Affiliations:** 1 Department of Emergency and Critical Care Medicine, Saiseikai Central Hospital, Tokyo, JPN; 2 Department of Emergency and Critical Care Medicine, Japanese Red Cross Musashino Hospital, Musashino, JPN

**Keywords:** acute poisoning, boric acid, continuous hemodiafiltration, drug intoxication, hemodialysis

## Abstract

We describe an 88-year-old female with chronic kidney disease (CKD) stage 3a (baseline serum creatinine, 1.1 mg/dL) who presented with vomiting and diarrhoea after accidentally ingesting 6 g of a boric acid solution intended for eye irrigation. On admission, her blood pressure was 95/49 mmHg, and physical examination revealed mild facial and chest erythema. As the ingested dose exceeded the toxic threshold and the patient exhibited potential for hemodynamic instability, continuous hemodiafiltration (CHDF) was initiated at 16 hours post-ingestion with the following parameters: blood flow rate, 100 mL/min; dialysate flow rate, 500 mL/h; and filtration rate, 500 mL/h. Mental status and skin condition improved, and CHDF was discontinued after 48 hours. By hospital day 45, the serum creatinine level returned to baseline, and chronic kidney function remained stable. Although the ingested dose was below the lethal range (15-20 g), impaired renal clearance likely prolonged systemic exposure, resulting in toxicity. This case suggests that early CHDF can be advantageous in patients with CKD experiencing boric acid poisoning, particularly when hemodynamic instability precludes intermittent hemodialysis, and can facilitate the prevention of renal function deterioration.

## Introduction

Boron (B), the fifth element in the periodic table, is ubiquitous in nature and widely present in soil, rock, surface water, and seawater in the form of boric acid and inorganic borates. Borates and boric acid have been used for many years in medical, industrial, and household settings. Currently, the primary routes of human exposure to boron are food (e.g., fruits, vegetables, nuts), water, and incidental exposure. Excessive ingestion of boric acid can cause toxicity involving the gastrointestinal tract, central nervous system, skin, and kidneys. Elimination of borates from the blood is largely by excretion (>90%) of the administered dose via the urine, regardless of the route of administration [1]. Because boric acid has a small molecular weight, low volume of distribution, and long half-life, haemodialysis is considered an effective treatment for boric acid poisoning [2,3]. In chronic renal failure, drug poisoning involving renally excreted drugs tends to become severe; in cases with unstable hemodynamics, continuous hemodiafiltration (CHDF) is recommended over intermittent hemodialysis. However, to our knowledge, no detailed case reports have described boric acid poisoning in patients with chronic kidney disease (CKD) or subsequent CKD exacerbation. Here, we present a case of acute boric acid poisoning in a patient with pre-existing CKD successfully treated with CHDF.

## Case presentation

An 88-year-old female with CKD stage 3b (baseline serum creatinine, 1.1 mg/dL) presented to the emergency department with chief complaints of vomiting and diarrhoea. She reported accidental ingestion of 6 g of a boric acid solution intended for eye irrigation, approximately 10 hours before admission. The following were her vital signs on arrival: Glasgow Coma Scale score [4], E3V4M6; SpO₂, 96% on room air; respiratory rate, 20 breaths/min; blood pressure, 95/49 mmHg; and heart rate, 92 beats/min. Physical examination revealed nausea, diarrhoea, and mild facial and chest erythema. The diarrhoea was watery and muddy (Bristol stool type 7); however, it did not exhibit greenish-yellow stools, typically observed in boric acid poisoning. The progression of laboratory findings from admission is summarized in Table 1.

**Table 1:** Laboratory parameters during hospitalization. Improvements in renal function were observed on Day 1, Day 3, and Day 45 based on Blood Urea Nitrogen, Creatinine, and estimated Glomerular Filtration Rate after initiating CHDF on Day 1. The progression of anemia and thrombocytopenia on the third day was thought to be caused by multiple factors, including dilution due to infusion and mechanical destruction by CHDF. CHDF: continuous hemodiafiltration.

	Reference range	Day 1	Day 3	Day 45
Blood Urea Nitrogen (mg/dL)	8.0-20	50	18	14
Creatinine (mg/dL)	0.46-0.79	2.42	0.95	0.77
Estimated Glomerular Filtration Rate (mL/min/1.73m^2^)	>60	15.1	42.0	52.8
Aspartate Aminotransferase (U/L)	13-30	35	49	17
Alanine Aminotransferase (U/L)	7.0-23	14	21	8
Albumin (g/dL)	4.1-5.1	3.5	3.0	3.1
White Blood Cell (/μL)	3.3-8.6×10³	17900	8300	8000
Hemoglobin (g/dL)	13.5-17.0	10.3	8.0	10.1
Platelet (/μL)	15.8-34.8×10⁴	247000	118000	297000
Sodium (mmol/L)	138-145	138	140	140
Potassium (mmol/L)	3.6-4.8	4.4	3.8	4.4
Chlorine (mmol/L)	101-108	106	106	106

The patient ingested 6 g of boric acid, exceeding the reported toxic dose for adults, and presented with the described findings, leading to a diagnosis of acute boric acid poisoning. However, the diagnosis was partially speculative due to the absence of measured serum boric acid levels. Due to a systolic blood pressure in the 90 mmHg range, the patient was admitted to the intensive care unit for close monitoring. CHDF was initiated immediately after admission and 16 hours after ingestion. The settings were: blood flow rate 100 mL/min, dialysate flow rate 500 mL/h, and filtration rate 500 mL/h. By hospital day three, blood urea nitrogen and serum creatinine had decreased to 18 mg/dL and 0.95 mg/dL, respectively. With improvement in consciousness and cutaneous symptoms, CHDF was discontinued approximately 48 hours after initiation. She subsequently required no further CHDF, and her condition remained stable at both the 7th and 14th days. By hospital day 45, serum creatinine had improved to 0.77 mg/dL and estimated glomerular filtration rate (eGFR) to 52.8 mL/min/1.73 m², returning to baseline levels. No worsening of CKD was observed.

## Discussion

Emergency medical transports related to suspected medication overdose among individuals aged ≥60 years have gradually increased from 2020 to 2022 in Japan [5]. According to the Japan Poison Information Centre, the number of inquiries concerning accidental ingestion of slime containing boric acid has also increased in recent years, 153 cases in 2022 and 173 cases in 2023 [6]. In elderly patients with chronic renal failure, even small doses of toxic substances can cause severe symptoms due to reduced renal clearance [7-9].

To date, however, there have been no documented cases of boric acid poisoning in CKD patients. The lethal dose of boric acid in adults is reported to be 15-20 g [10]. Although the ingested dose in this case (6 g, or 120 mg/kg) was below that threshold, impaired renal function likely contributed to prolonged systemic exposure and the potential for severe symptoms. Supporting this hypothesis, a comparative study of 17 patients undergoing maintenance haemodialysis and 467 individuals with normal renal function found that baseline serum boron concentrations were significantly higher in dialysis patients [11]. Additionally, a study of 784 boric acid poisoning cases reported a mean intake of 3.2 g (range 0.1-55 g) in symptomatic patients, compared with 0.9 g (range 0.01-88.8 g) in asymptomatic patients [12]. These findings suggest that in CKD patients, serum boric acid levels can rise more markedly than in those with normal renal function, and ingestion exceeding approximately 3 g may lead to severe toxicity.

Most prior reports of boric acid poisoning treated with extracorporeal therapy have utilised intermittent haemodialysis (HD). However, successful cases using CHDF have been described in patients with circulatory failure. In our patient, hypotension secondary to vomiting and diarrhoea indicated a risk of haemodynamic instability during HD; thus, CHDF was selected. CHDF was performed with the following prescription: blood flow rate 100 mL/min, dialysate flow rate 500 mL/h, and ultrafiltration rate 500 mL/h. Two factors informed this prescription. First, Japanese insurance regulations limit CHDF prescription volume to 18 L/day. Second, these settings were based on previous case reports. Among Japanese and English case reports that clearly described ingestion circumstances and dialysis parameters, only one by Oonishi et al. met these criteria [13]. Their case involved an 80-year-old woman (body weight 42 kg) treated 24 hours after ingestion with CHDF (blood flow rate 80 mL/min, dialysate 500 mL/h, filtration 800 mL/h), achieving boric acid clearance of 24.1 mL/min and clinical recovery.

In our case, boric acid concentration was not measured because testing was unavailable commercially. The optimal CHDF prescription for boric acid poisoning remains unclear, and further studies are needed. Although the limitations include the inability to establish a clear causal relationship that early use of CHDF prevents worsening of CKD, this case adds valuable information by describing the ingested dose, timing, and dialysis parameters, contributing to future efforts to standardise management.

Pathological studies of acute boric acid poisoning have revealed acute tubular necrosis (ATN) [14,15]. Because ATN is a known risk factor for CKD progression, boric acid poisoning may exacerbate pre-existing renal disease [16,17]. Previous case reports of boric acid poisoning have rarely documented baseline renal function, leaving renal prognosis in CKD patients uncertain. In our patient, renal function did not deteriorate at three months, suggesting that early initiation of dialysis may have prevented CKD progression.

## Conclusions

This case of boric acid poisoning in a patient with pre-existing CKD resulted in a favourable short to mid-term outcome without renal deterioration and suggests potential benefit and supports consideration of early extracorporeal therapy. Early CHDF may represent an appropriate treatment option for chronically ill patients with unstable haemodynamics. Given the limited evidence base, the optimal timing, dose, and stopping criteria for extracorporeal therapy in boric acid poisoning, especially in CKD, remain to be established. Accumulation of further cases and prospective data is warranted to define practical initiation thresholds and patient-centred endpoints.
